# The investigation of levels of endothelial cell-specific molecule, progranuline, clusterin, and human epididymis protein 4 in the differential diagnosis of malignant pleural effusions

**DOI:** 10.1097/MD.0000000000032471

**Published:** 2022-12-30

**Authors:** Soner Demirbas, Fatma Hümeyra Yerlikaya, Sebnem Yosunkaya, Ummugulsum Can, Korkmaz Celalettin

**Affiliations:** a Department of Chest Diseases, Meram Faculty of Medicine, Necmetin Erbakan University, Konya, Turkey; b Department of Biochemistry, Meram Faculty of Medicine, Necmettin Erbakan University, Konya, Turkey; c Department of Biochemistry, Konya Training and Research Hospital, Konya, Turkey.

**Keywords:** clusterin, endothelial cell-specific molecule, human epididymis-4, malignant pleural effusion

## Abstract

Progranulin (PGRN), endothelial cell-specific molecule-1, clusterin (CLU), and human epididymis protein 4 (HE-4) are novel proteins reported to have diagnostic and prognostic potential in lung cancer. Here, we aimed to identify the markers with high sensitivity and specificity in distinguishing malignant pleural fluids from other pleural fluids.

**Methods:**

This prospective, descriptive study was conducted at a medical faculty hospital between 2016 and 2019. The study population consisted of 90 patients <18 years of age with pleural effusion (PE). Levels of pleural fluids of PGRN, endothelial cell-specific molecule-1, CLU, and HE-4 were measured with enzyme-linked immunosorbent assay kits under the manufacturer’s manual.

**Results:**

Of 90 patients, 54 were men, and 36 were women (mean age 65 ± 16 years). Of pleural fluids investigated, 23 (25%) and 67 (74%) were transudates and exudates, respectively. Of exudates, while 27 (40%) and 19 (28%) were parapneumonic PE and tuberculous PE, respectively, 20 (29%) were malignant pleural effusion (MPE). Levels of all biomarkers in exudate fluids were found significantly higher than those of transudate fluids. CLU, HE-4, and PGRN levels in MPE were also found significantly higher than benign fluids (*P* < .05). Cutoff values were achieved by receiver operating characteristics analysis for CLU, HE-4, and PGRN to distinguish between malignant and benign groups. For diagnosis of MPE, the sensitivity and specificity values were found as 0.66 and 0.67 for a cutoff value of CLU of 18.29 mg/L (*P* = .00), as 0.76 and 0.76 for a cutoff value of HE-4 of 9.33 mg/L (*P* = .00), and as 0.66 and 0.67 for a cutoff value of PGRN of 105.91 mg/L (*P* = .001).

**Conclusion:**

HE-4 having high sensitivity and specificity can be a potential diagnostic marker in distinguishing between malignant and benign effusions, and these findings can constitute a basis for future research.

## 1. Introduction

The first step in the differential diagnosis of pleural effusion (PE) is to determine whether the effusion is transudative or exudative. Light criteria are used to distinguish between transudates and exudates.^[1]^ Common causes of exudative pleural effusion (EPE) are malignancy, parapneumonic effusion, and tuberculosis (TB).^[2]^ In various studies, malignant pleural effusions (MPE) are shown as one of the most common causes of exudate fluids (42%–72%).^[3]^ MPE can be due to the complication of any malignancy; however, MPE incidence ranges between 7% and 23% in patients with lung cancer.^[4]^ Malignant effusions in lung cancer not only impairs the quality of life of patients but indicate a poor prognosis, as well.^[5]^ Although PEs are malignant in most lung cancer patients, benign effusions may also develop during the diagnosis or the clinical course of the condition. In a study by Porcel et al, benign causes of PEs in those with lung cancer were shown to consist of up to 20% of all effusions.^[6]^ Therefore, the diagnosis of malignant effusion in lung cancer is of vital importance in terms of the early initiation of therapy and management of symptoms. Although critical, accurate diagnosis of MPE still remains a difficult procedure. The cytological examination is the traditional and standard method for its diagnosis; however, its sensitivity varies between 30% and 60%.^[7]^ Additionally, the efficacy of sequential blind pleural biopsies has been asserted to be limited, and so blind biopsies of MPEs are diagnostic in only 20% of patients with negative cytology of malignant effusion.^[8]^ Even though it is the “gold standard” in the diagnosis of malignancy,^[1]^ the thoracoscopic pleural biopsy is more invasive and costly. For these reasons, medical science now keeps seeking an answer to the question of “Can PEs be evaluated with less invasive and inexpensive methods?”

MPE has traditionally been associated with the obstruction of parietal pleural stomata or the occlusion of lymph vessels in mediastinal lymph nodes. However, such features as the lymphatic block, inflammation, increased angiogenesis, and vascular leakage are deprived of referring fully to MPE.^[1,9]^ Vascular endothelial growth factor-A (VEGF-A) is a critical cytokine provoking the extravasation and formation of MPEs. Even so, VEGF is not the only vasoactive cytokine involved in MPE. Other molecules provoking the formation of MPEs are pro-inflammatory cytokines, such as tumor necrosis factor (TNF), chemokine (C-C motif) ligand 2 (CCL2), and osteopontin (OPN) Osteopontin (OPN), also known as bone sialoprotein I (BSP-1 or BNSP).^[10]^

Endothelial cell-specific molecule-1 (ESM-1) is a 50 kDa soluble proteoglycon composed of a mature polypeptide of 165 amino acids and associated with the endothelial cells expressed by the renal and pulmonary endothelium. ESM-1 was originally cloned from a human endothelial complementary DNA by Lassalle et al,^[11]^ and in the later period, ESM-1 expression was found out to be associated with lung, kidney, and breast cancers.^[12–15]^

Progranulin (PGRN) is a glycoprotein with 593 amino acids, the mRNA of which is expressed both in vitro and in vivo by many epithelial cells. PGRN levels have been identified as a new parameter determining the rate of epithelial proliferation leading to tumor growth in some cell lines. This enables PGRN to play a critical role in epithelial proliferative homeostasis and may lead to important consequences in the development and growth of carcinomas.^[16]^

Clusterin (CLU) exists widely in various tissues and organs and is involved in several biological processes, including fluid transport, cell apoptosis, and cell adhesion, etc.^[17]^ The abnormal expression of CLU protein is reported to be associated with cardiovascular diseases, inflammatory diseases, and tumorigenesis.^[18,19]^ In most of the clinical studies, it was shown that CLU is an indicator of poor prognosis associated with tumor recurrence and metastases, including lung cancer, and that secretory clusterin (sCLU) is associated with increased survival and proliferation of non-small cell lung cancer (NSCLC), thus leading to poor prognosis.^[20]^

The protein of human epididymis protein 4 (HE-4) is encoded by a gene located on chromosome 20q12-13.1 and is known as a precursor of the human epididymis protein. HE-4 is frequently overexpressed in ovarian cancers; however, other expressions are also found in lung, endometrial, breast adenocarcinomas, and mesotheliomas, and to a lesser degree, in the gastrointestinal, kidney, and transitional cell carcinomas.^[21]^ In a meta-analysis including a total of 1412 patients from eight studies, it was reported that higher HE-4 expression was associated with poorer overall survival in Asian lung cancer patients although no significant relationship was found in Caucasian lung cancer patients.^[22]^ Another meta-analysis including a total of 16 studies (1756 lung cancer and 1446 controls) also revealed that HE-4 had a moderate diagnostic accuracy rate for lung cancer; the analysis of HE-4 should be interpreted in parallel with the clinical findings and results of other traditional tests; more studies are needed to meticulously evaluate the diagnostic accuracy of HE-4 for lung cancer.^[23]^

For MPEs, a standard biomarker has yet to be identified. Ideal biomarkers should be cost-effective and easily measurable, sensitive, and specific to the disease investigated, and contribute to decision-making for the diagnosis.^[24]^ In our study, it was investigated whether ESM-1, PGRN, CLU, and HE-4 can be utilized in PE to differentiate between malignant patients and nonmalignant diseases causing PEs.

## 2. Materials and Methods

### 2.1. Study participants and samples

Ninety patients with PE admitted to the department of chest diseases of a university hospital between 2016 and 2019 were prospectively enrolled in the study. On admission, pleural fluids were drawn from each patient through the thoracentesis and centrifuged at 3000 rpm for 10 minutes. The supernatants were frozen and stored at −80° C until analysis. In order to investigate PGRN, ESM-1, CLU, HE-4 concentrations in Pes, the levels of concentrations were measured for PGRN with the human progranulin immunoassay kit (Boster Biological Technology Co. Ltd. Pleasanton, CA), for ESM-1 with the enzyme-linked immunosorbent assay (ELISA) kits (Elabscience Biotechnology Co., Houston, TX), for CLU (Guandong Science and Technology Industrial Park, WuHan, Hubei, China), and HE-4 (YH Biosearch Lab., Jinhu Road, Pudong District, Shanghai, China). The levels were evaluated using the immunoassay kits under the manufacturers’ instructions. The biochemists assessing all samples were blinded to perform the assays, and all levels were determined in a duplicated form. The present study was conducted after written informed consent was obtained from all participants. The study protocol was approved by the ethics committee of the institution.

### 2.2. Diagnostic criteria

The determination of PE etiology for each patient was performed, based on the clinical presentation, diagnostic test results, and response to the treatment. Based on Light’s criteria for exudate fluids, while levels of pleural fluid/serum protein and pleural fluid/serum lactate dehydrogenase (LDH) were defined as >0.5 and >0.6 respectively, level of pleural fluid LDH was defined as >2/3 of the upper limit of the reference range for serum LDH. If malignant cells were observed on cytological examination or in the pleural biopsy specimen, MPE was diagnosed. As consistent with the design of the study, only malignant effusions associated with lung cancer were recorded. Parapneumonic effusion (PPE) was defined as the patient’s novel fever, purulent sputum, and the presence of pulmonary infection with pneumonic infiltration on chest radiography, responding to antibiotic treatment. However, tuberculous pleural effusion (TPE) was diagnosed through the pathological confirmation by pleural biopsy or the positivity of biopsy material or TB culture of pleural fluid in those with lymphocyte predominance and high level of adenosine deaminase (>40 IU/L) in the pleural fluid.

### 2.3. Statistical analysis

The data detected in the study are presented at median and interquartile intervals. To compare the differences between the groups, the Kruskal–Wallis or the Mann–Whitney *U* test was used for nonparametric variables. The receiver operating characteristics (ROC) curves were analyzed to determine the optimum cutoff values and to compare the diagnostic accuracy of the markers. The cutoff value for each marker was determined based on the best diagnostic efficiency obtaining the balance between the sensitivity and specificity. If a *P* value was below 0.05, the difference was considered statistically significant. The statistical analysis was performed using the Statistical Package for the Social Sciences software version 18.0 for Windows (SPSS Inc., Chicago, IL).

## 3. Results

### 3.1. Patients’ characteristics

A total of 90 patients, including 21 malign PE in primary lung carcinomas group and 69 in the benign PE group were enrolled in the study. Among lung cancer patients, 11 adenocarcinomas, eight squamous cell carcinomas, and two small cell carcinomas were detected. There were 19 TPE, 27 PPE, and 23 transudate PE in the group with benign PE. Of 90 study participants, 54 (69%) were male, and the age level was detected not to be significantly different between malignant and benign PE groups (*P* > .05).

However, the age level of the patients with TPE was found to be significantly lower, compared with the other groups (*P* = .001). The clinical features of each group are summarized in Table 1.

**Table 1:** Clinical characteristics and diagnosis of 90 patients in the study.

Diagnosis	Patients (n)	Male/female (n)	Mean age (yr)	Age range (yr)
Malignant PE	21	15/7	67.8	32–84
Parapneumonic PE	27	18/9	72.6	48–85
Tuberculosis PE	19	11/8	55.3	25–75
Transudate PE	23	10/13	66.4	48–86
Total	**90**	**54/37**	**65.52**	**25–86**

PE = pleural effusion.

### 3.2. Concentrations in each group

The pleural fluid levels of ESM-1, HE-4, and PGRN were significantly higher in the exudative PE group, compared with the transudate group (*P* < .05). Even so, there was a borderline significance for CLU (*P* = .05) (Table 2). Additionally, the levels of CLU, HE-4, and PGRN were also found to be significantly higher than nonmalignant fluid levels among MPE patients (*P* < .05). Average concentrations of CLU, ESM-1, HE-4, and PGRN in patients with MPE, TPE, PPE, and transudate are shown in Table 2.

**Table 2:** Mean concentrations of biochemistry in patients with lung cancer, tuberculous pleurisy, parapneumonic pleural effusion, and transudate.

	Transudate	Exudate		
	Nonmalignant			Malignant
	Transudate	Parapneumonic	Tuberculosis	Malignant
	Mean ± SD	Mean ± SD	Mean ± SD	Mean ± SD
CLU	14.31 ± 1.33	17.64 ± 1.91	12.65 ± 1.70	24.96 ± 2.61
ESM-1	631.01 ± 39.57	952.59 ± 65.76	802.80 ± 69.50	853.96 ± 73.54
HE-4	5.56 ± 0.67	8.49 ± 0.82	6.31 ± 0.73	15.31 ± 1.59
PGRN	65.37 ± 7.22	380.33 ± 108.25	133.97 ± 60.11	317.06 ± 80.38

CLU = clusterin, ESM-1 = endothelial-specific molecule-1, HE-4 = human epididymal protein-4, PGRN = progranulin, SD = standard deviation.

**Figure 1.**
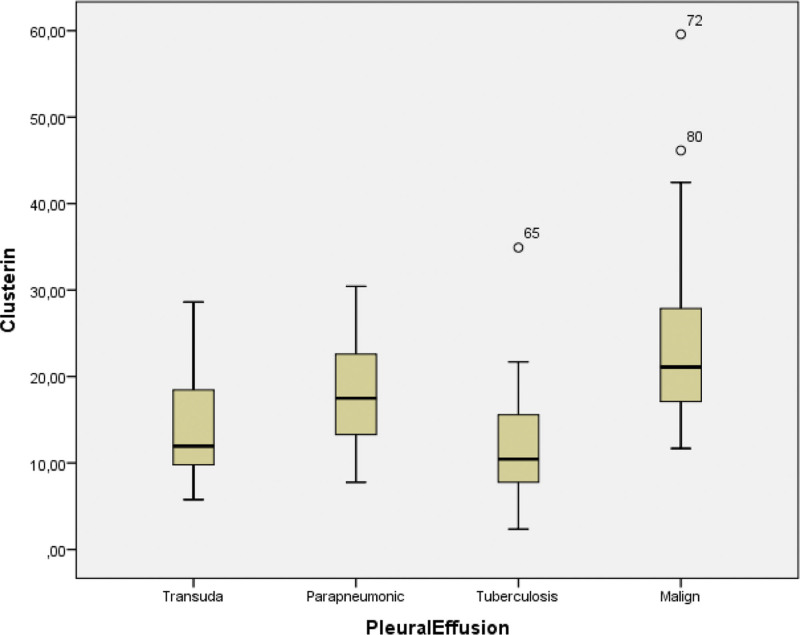
Concentrations of clusterin in patients with lung cancer, tuberculosis, parapneumonic pleural effusion, and transudate.

The values of CLU were measured as 12.65, 17.64, 24.96, and 14.31 ng/mL for TPE, PPE, MPE, and transudate, respectively (Table 2). The level of CLU in the pleural fluid of the patients with lung cancer was significantly higher than that of PPE, TPE, and transudate groups (*P* < .001) (Fig. 1).

**Figure 2.**
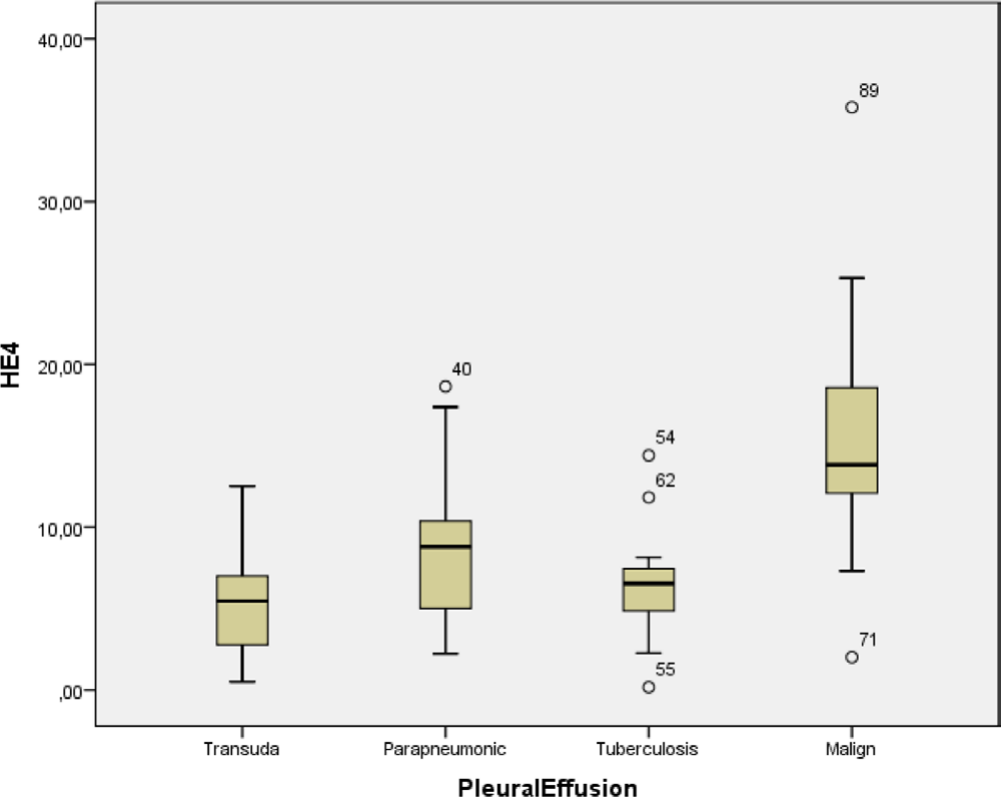
Concentrations of HE-4 in patients with lung cancer, tuberculosis, parapneumonic pleural effusion, and transudate. HE-4 = human epididymal protein-4.

HE-4 of the patients with MPEs were significantly higher than that of other groups (*P* < .001) (Fig. 2).

**Figure 3.**
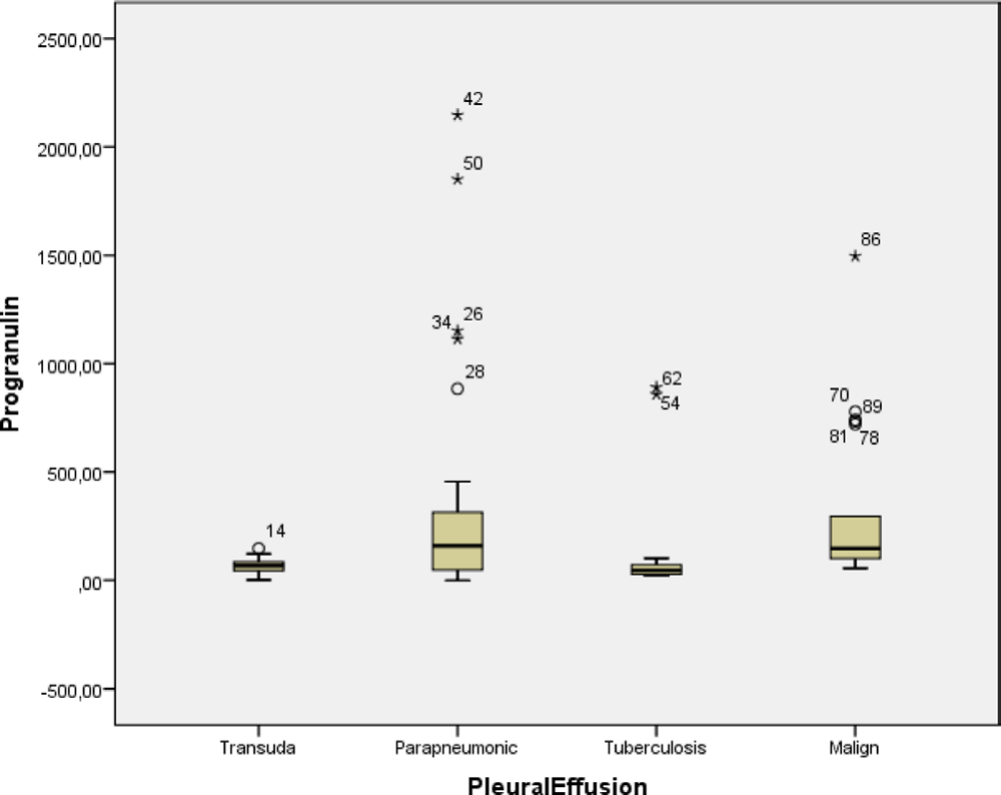
Concentrations of progranulin in patients with lung cancer, tuberculosis, parapneumonic pleural effusion, and transudate.

Mean fluid levels of PGRN were found as 317.06, 133.97, 380.33, and 65.37 ng/mL for MPE, TPE, PPE, and transudate groups, respectively (Table 2). The level of PGRN among the patients with PPE, MPE, and TPE was higher than that of the transudate group (*P* < .001) (Fig. 3). The highest average PGRN concentrations were obtained from those with PPE (Fig. 3). PGRN levels of patients with lung cancer were significantly higher than those of the patients with TPI and transudate ((*P* < .001). However, no significant difference was found between MPE and PPE in terms of PGRN (*P* = .747).

**Figure 4.**
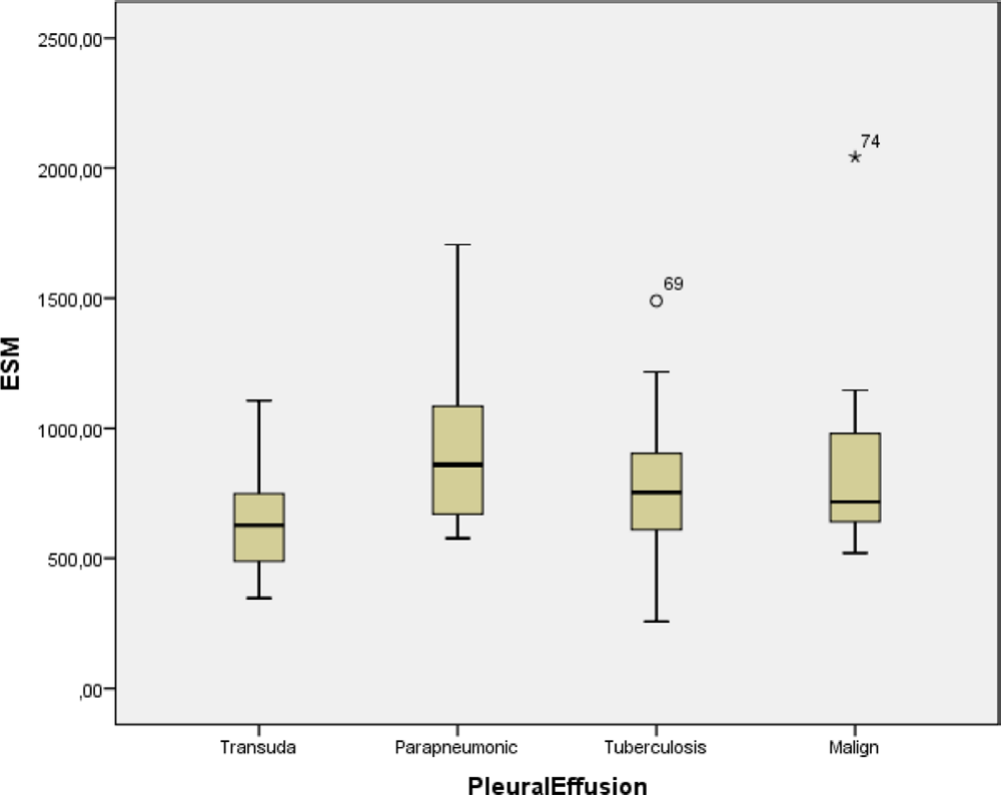
Concentrations of ESM-1 in patients with lung cancer, tuberculosis, parapneumonic pleural effusion, and transudate. ESM-1 = endothelial-specific molecule-1.

Mean concentrations of ESM-1 were obtained as 853.96, 802.80, 952.59, and 631.01 ng/mL, for MPE, TPE, PPE, and transudate groups, respectively (Table 2). ESM-1 level of lung cancer patients was significantly higher than that of the transudate group (*P* = .001, *χ*^2^ 0015.487); however, ESM-1 was not significantly different from that of the patients with TPE and PPE (*P* = .766, *χ*^2^ = 0.089 and *P* = .291, *χ*^2^ = 2.470) (Fig. 4).

**Figure 5.**
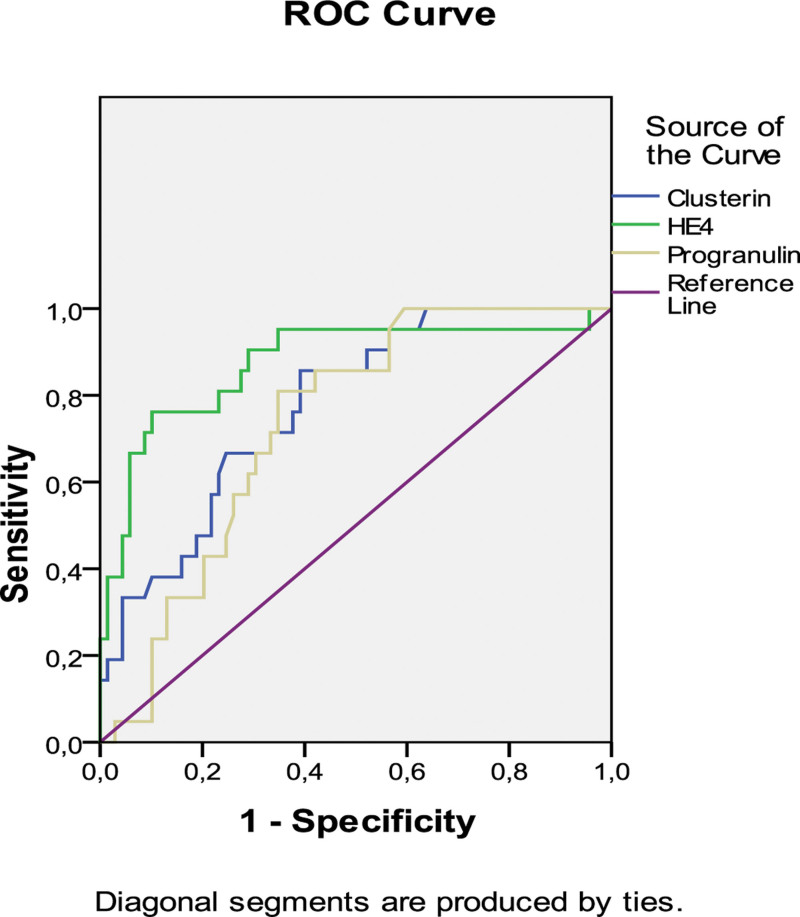
ROC curves for malignant and nonmalignant liquid separation. ROC = receiver operating characteristics.

The cutoff values for CLU, HE-4, and PGRN in distinguishing between malignant and nonmalignant fluids were determined using ROC curves (Fig. 5).

Given the cutoff value as 18.29 mg/L for CLU in the diagnosis of MPE, the sensitivity and specificity were calculated as 0.66 and 0.67, respectively. The area under the curve (AUC) was also found as 0.776 [95% confidence interval (CI), 0.673–0.879] (*P* = .00). Considering the cutoff value for HE-4 as 9.33 mg/L, the sensitivity and specificity rates were found to be 0.76 and 0.76, respectively; AUC was 0.874 (95% CI, 0.775–0.972) (*P* = .00). Given the cutoff value as 105.91 mg/L for PGRN, the sensitivity and specificity rates were calculated as 0.66 and 0.67 respectively, and AUC was 0.733 (95% CI, 0.629–0.836) (*P* = .001).

## 4. Discussion

In our study, we investigated the pleural fluid levels of ESM-1, PGRN, CLU, and HE-4, considered being a useful diagnostic approach in distinguishing between pleural fluids, especially malignant fluids from exudate fluids. We revealed that all of the biomarkers studied in the study had higher levels in the exudative fluid than those in transudative pleural fluid. However, grouping PEs as transudates and exudates does not provide a disease-specific diagnosis. In previous studies, various researchers, including Light,^[1]^ suggested focusing on the research for the identification of specific pleural disease markers. In the present study, the levels of HE-4, CLU, and PGRN were revealed to be higher in malignant fluids. However, lung cancer was differentiated in merely the pleural fluid level of HE-4 from benign PEs with marked specificity and sensitivity. When the cutoff value was considered 9.33 mg/L, both sensitivity and specificity of HE-4 were found be 76% for the diagnosis of MPE, and AUC was 0.874 (95% CI, 0.775–0.972) (*P* < .001). In previous studies, the expression of HE-4 has also been shown in lung cancer cell lines.^[25,26]^

In light of the literature, there are so few studies investigating the diagnostic value of HE-4 in malignant PE among those with lung cancer. In a study performed by Lv et al, HE-4 protein in PE of 60 patients with lung cancer was compared with 56 patients with benign lung disease. In the study by Lv et al, as consistent with our findings, the HE-4 level was significantly higher among lung cancer patients than that of those with benign lung disease (*P* = .001). With the use of an optimal threshold of 652.2 pmol/L, the HE-4 level distinguished malignant lung cancer from benign lesions with a sensitivity of 78.3% and specificity of 75.0%. Additionally, patients with lung adenocarcinoma demonstrated significantly higher levels of HE-4 than those with squamous cell carcinoma and small cell carcinoma.^[27]^ In another study conducted in 88 patients with different types of PE by Elsammak et al, both serum HE-4 levels and HE-4 levels of PE were detected to be significantly higher in the patients with lung and extrapulmonary malignant effusions than those with transudative or nonmalignant exudative effusion. In the study, a cutoff value of 1.675 pmol/L was revealed in pleural fluid with a diagnostic sensitivity of 85.3% and a specificity of 90.7%, predicting malignant PEs.^[28]^

In the study where the serum samples obtained from 100 patients with lung cancer, 57 patients with benign lung disease, and 274 healthy controls by Choi et al, HE-4 levels were found to be significantly higher in the patients with lung cancer than those with benign lung disease and healthy controls (*P* < .0001); the area under the ROC curve was 0.84 for HE-4 (95% CI, 0.78–0.89; *P* < .001). In the same study, HE-4 levels were observed to increase significantly in all different histological subgroups of lung cancer, and also among those with the smallest tumors (*P* = .002).^[29]^ In another study, when compared with small-cell, squamous, and large-cell lung cancers HE-4 tissue expression was shown to be the highest in adenocarcinomas of the lung.^[30–33]^ In our study, while no significant difference was found between the types of malignancies, most of the malignant fluids were detected in the patients with adenocarcinomas, and the number of small cell carcinomas was seen to be very low. For this reason, we may have found no significant difference between HE-4 levels of the fluids. Finally, in the study by Galgano et al, it was demonstrated that of 47 patients with mesothelioma, 28 had HE-4 expression somewhat.^[30]^

In the study by Scherpereel et al, a significant increase was observed in the serum levels of ESM-1 in patients diagnosed with lung cancer.^[34]^ In mouse tumor models, the serum levels of ESM-1 were found to be correlated with the size of tumors. It is a known fact that ESM-1 is mainly produced by vascular endothelial cells in humans.^[11,35]^ It seems reasonable to hypothesize that increased serum levels of ESM-1 reflect the activation of the tumor vascular bed and perhaps tumor proliferation in patients with lung cancer. However, it is too early to consider that ESM-1 in circulation may be a beneficial marker for cancers in humans. As soon as we are concerned, there is no study investigating malignant pleural fluids in the differential diagnosis of malignant PE. While the level of ESM-1 was significantly higher among the patients with lung cancer than that of those in the transudate group, ESM-1 concentrations to help distinguish other exudative fluids (TB and parapneumonic) from MPE could not be obtained in our study.

PGRN is a multifunctional glycoprotein playing a role in the formation, development, inflammation, and repair of tumors.^[36]^ To the best of our knowledge, there is no previous study evaluating the differential role of PGRN in pleural fluids. In our study, PGRN levels of parapneumonic, malignant, and tuberculosis PEs were higher than the transudate level. PGRN level was also found to be significantly higher in patients with lung cancer, compared to TB effusions; even so, no significant difference was found in distinguishing between MPE and PPE. Among the exudates, the highest level of PGRN was observed in PPE.

CLU is a secretory glycoprotein and has been stated to be upregulated in various human cancers, including breast, colon, prostate, and lung cancers.^[37]^ Immunohistochemistry staining results also revealed that CLU is highly expressed in vascular cells (including endothelial cells and smooth muscle cells) in lung cancer tissues, indicating that CLU may be involved in tumor angiogenesis.^[37]^ In the meta-analysis of 26 immunohistochemical studies, where the prognostic role of CLU was investigated in multiple malignant neoplasms, CLU has been reported to be a potential biomarker for both recurrence-free survival and disease-free survival.^[38]^ Evidence also suggests that CLU is overexpressed in patients with metastatic tumors and experimental metastasis models. Specifically, CLU has been shown to play a role in the antiapoptotic capacities, the enhancement of treatment resistance, and the induction of epithelial-mesenchymal transition, all of which are associated with cancer metastasis. In a study, it has been reported that inhibition of CLU increases the cytotoxic effects of chemotherapeutic agents and improves the survival of stage-III and IV cancer patients.^[39]^ In our study, CLU was detected to be higher levels in MPEs, compared to transudate and nonmalignant fluids; however, the specificity and sensitivity rates were found to be lower in distinguishing between malignant and nonmalignant fluid.

## 5. Conclusion

The level of HE-4 was found to be higher in patients with primary lung carcinomas, compared with transudative fluids and other exudative fluids. HE-4 could be a potential marker in detecting tumors in the pleural fluids of primary lung carcinomas. However, we consider that to benefit as a diagnostic tumor marker in the clinical setting, HE-4 should be investigated further in tissue expression and at serum levels in benign and malignant lung diseases, as well as the substrates of different lung cancers.

## 6. Limitations

The present study was performed only in a single province, and the number of study population was limited. For this reason, more comprehensive studies with larger populations are needed to shed light on the entity.

## Author contributions

**Investigation:** Fatma Hümeyra Yerlikaya, Sebnem Yosunkaya, Soner Demirbas, Ummgülsüm Can.

**Methodology:** Ummgülsüm Can.

**Resources:** Soner Demirbas.

**Supervision:** Soner Demirbas.

**Writing – original draft:** Soner Demirbas.

**Writing – review & editing:** Celalettin Korkmaz.
